# Fungal networks serve as novel ecological routes for enrichment and dissemination of antibiotic resistance genes as exhibited by microcosm experiments

**DOI:** 10.1038/s41598-017-15660-7

**Published:** 2017-11-13

**Authors:** Rashid Nazir, Ju-Pei Shen, Jun-Tao Wang, Hang-Wei Hu, Ji-Zheng He

**Affiliations:** 1 State Key Laboratory of Urban and Regional Ecology, Research Centre for Eco-Environmental Sciences, Chinese Academy of Sciences, Beijing, 100085 China; 2 Department of Environmental Sciences, COMSATS Institute of Information Technology, Abbottabad, 22060 Pakistan; 3 Faculty of Veterinary and Agricultural Sciences, The University of Melbourne, Parkville, Victoria, 3010 Australia

## Abstract

Antibiotic resistance genes (ARGs) in the environment and their subsequent acquisition by clinically important microorganisms are a serious concern. However, the spread of environmental ARGs remain largely unknown. We report, for the first time, the involvement of soil fungi in the distribution of bacteria with ARGs via soil microcosms. qPCR assay detected unique ARGs specifically found in the mycosphere of different fungi. Interestingly, the taxonomically and ecologically different fungi exerted different selection pressures on ARGs originating from the same source. Test fungi supported different antibiotic resistance bacteria enriched in the mycosphere and even transported to distant places. The relative abundance of the *tnpA* gene decreased, for manure, along mycelial networks of all fungi. While the fungal strain NFC-5 enriched the *intI1* gene more, opposite to two other fungi at the migration front compared with the inoculation point for both sources. Such data indicate the differential effect of different fungi to facilitate horizontal gene transfer potential under fungal selection pressure. Our study provides the evidence that fungi can contribute ARGs, host bacterial diversity and abundance, and such interactive microbial consortia have the potential to disseminate the resistance determinants from one place to another, thus increasing the ARGs exposure risk to humans.

## Introduction

Over the last several decades, there has been an enormous increase in antibiotic use in clinics as well as in agriculture and animal husbandry. In parallel, microbes have developed a range of strategies (cumulatively called resistome) to deal with these antibiotics, subsequently attaining multiple drug resistance via evolving antibiotic resistance genes (ARGs)^1^. As a result, not even a century after the discovery of the first antibiotic, we are facing a worldwide public health crisis as ARGs have been recognized as an emerging environmental contaminant^2^. Among different sources of ARGs, animal manure and wastewater treatment plants (WWTPs) have been recognized as important reservoirs for their environmental dissemination^3–6^. Along with enhanced abundance and diversity, environmental ARGs may transfer to humans through their persistence and spread patterns (e.g. via horizontal gene transfer (HGT) to antibiotic resistant bacteria (ARB) subsequently disseminating into the food chain another). The emerging spread of ARGs and their potential acquisition by pathogens is thus an obvious threat to human health. Therefore, it is essential to understand the dissemination mechanisms of ARGs in the environment as well as the potential factors contributing to the transfer.

As a result of drastically increased antibiotic use and multiple contamination sources, the antibiotic resistance development has been exponentially raised^2,5^. Bacterial evolution has greatly been shaped by their genome plasticity, leading to their adaptation to diversified ecosystems. Such bacterial ability, to exchange and rearrange genomic sequences gaining new traits, has been extensively demonstrated with antibiotic resistance^7^. In this regard, the fungal infested soil (i.e. the mycosphere defined here as the soil with fungal hyphal networks^8^) has recently been suggested to constitute a gene transfer arena^9^ where different genes, potentially including ARGs, are swapped across the resident microbial communities^10^. Though several papers are available regarding the bacterial role in the acquisition and dissemination of ARGs, other biotic factors such as plants and fungi have never been considered to play a role. Therefore, the role of fungi, the ultimate partner of bacteria, which are the host or carrier of ARGs in the soil, needs to be investigated in the context of antibiotic resistance.

In soil environmental settings, bacteria and fungi live together and even help each other mutually benefiting from their cumulative ecological success^11,12^. In an earlier study, populations of *Bacillus subtilis* containing a tetracycline resistance plasmid were maintained in sterilized and fresh mushroom compost at 37 °C, while the survival at a higher temperature (65 °C) was greater in fresh (probably having living hyphae) than in sterilized compost^13^. Some ARGs were recently found to increase in abundance and transferability in the field after manure application^14,15^, suggesting the ongoing antibiotic resistance selection which could also be exerted via soil fungi. Bacterial cells of *Pseudomonas aeruginosa* associated with polymicrobial biofilms of *P*. *aeruginosa* and *Aspergillus fumigatus* were found to be comparatively recalcitrant to antibiotics compared to monomicrobial biofilms, while the planktonic cells in mono and mixed microbial cultures showed no difference in antimicrobial profiles^16^. Very recently, the fungus *Candida auris* has been reported to be antibiotic resistant, causing the outbreaks of invasive infections in an unusual way^17^. The fungal aspect is therefore important in the antibiosis- scenario particularly when the bacteria-fungal consortium has recently been reported to decrease the fungal susceptibility to antibiotics applied in soil^12^. Additionally, the increased abundance and transferability of ARGs have already been correlated with antibiotic concentration, root exudates and soil abiotic attributes in specific environments like the rhizosphere^15^. There is, thus a pressing need to evaluate other biotic factors (including fungi specifically) during environmental antibiosis.

Soil fungi are known to enrich bacteria by harboring cryptic and nutrition assisting plasmids in their mycosphere^18^. Furthermore, soil colonizing fungi directly influence the bacterial communities^19^ and even transfer them from one place in the soil to another^8^. In the natural environment, because fungi can grow over large distances^20^, they may play an important role as vectors of ARB and ARGs. Particularly, nutrients provision^21^ and bacterial survival^22^ are preferentially influenced by soil fungi, underpinning their potential role in the distribution of ARGs via changing diversity and abundance of bacteria hosting the resistance determinants. Therefore, we hypothesized that growing mycelial networks (the mycosphere^8^) of soil fungi would affect the diversity and abundance of co-inhabiting ARB, while the distribution and dynamics of soil ARGs will ultimately be influenced by soil colonizing fungi. There is little known regarding the influence of fungi growing in soils (with manure and/or other contamination sources) on the abundance and transfer of ARGs and also the selection of ARB populations. This study was thus designed, in soil microcosms, to explore the following particular objectives: i) the influences of fungal colonization in soil on the abundance of ARGs and the community composition of culturable ARB; ii) the effect of different manure originating fungi on the ARGs and ARB from manure as well as wastewater contamination sources; iii) if fungi can transport ARGs and ARB from one place in soil to another via mycelial networks, using microcosms.

## Results

### Characteristics of the isolated fungal strains

**Figure 1:**
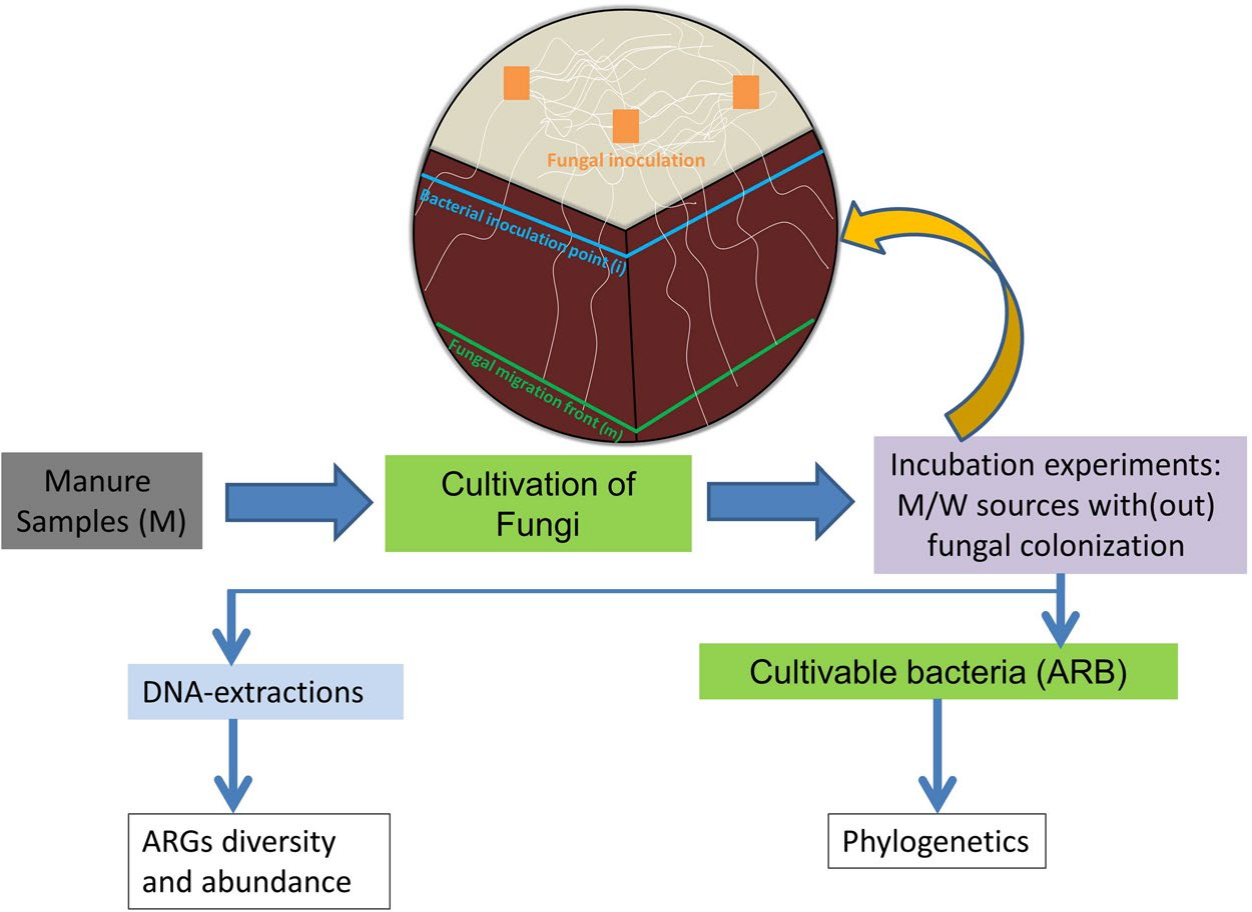
Schematic description of the soil microcosm construction and the experimental procedures used in this work. 3-comparmental Petri plate on top represents a microcosm with fungal growth ‘potato dextrose agar’ (PDA) in light colored top section; dark brown colored 2-sections represent soil; blocks in PDA section are the fungal inocula; white colored irregular lines represent fungal hyphae; the solid lines (blue and green) are the sampling points. Manure suspension was used to enrich and purify the fungal strains which were then inoculated to colonize the pre-sterilized soil in microcosms, where bacterial suspension was applied as inoculum (i.e. inoculation point (i)) either from manure or waste water adjacent to fungal front (<1 cm in soil at that moment). Fungus kept growing and reached till end of the Petri plate (i.e. fungal migration front (m)). Consequently, the sampling was done from different points (i and m) of the soil microcosm and processed for culture based and molecular analyses. M, microbial inoculum originating from manure; W, microbial inoculum originating from sludge/water of waste water treatment plant; ARB, antibiotic resistant bacteria; ARGs; antibiotic resistance genes.

The saprotrophic fungal strains were isolated (on PDA) from manure sample (M) collected from an animal farm. The cultivable fungal colonies were 2 × 10^5^ per g, out of which 16 strains were purified on the basis of colony morphology and growth characteristics (Table 1). The molecular identification via sequencing of the ITS region exhibited that the purified fungal strains were taxonomically diverse. ITS-gene sequences were blast analyzed against NCBI database and closest hits, on the basis of similarity i.e. ≥99%, were considered for fungal identification (Table 1). Based on soil colonizing characteristics, three of the purified fungi, namely NFC-5, NFC-14 and NFC-16 (members of the genus *Trichoderma* sp., *Schizophyllum* sp. and *Coprinellus* sp., respectively), with relatively high infestation rates (Table 1) were selected for soil microcosm studies (described in Fig. 1) to explore the effect of fungi on the distribution of ARB and ARGs. Such fungi are widespread and prevalent in a range of terrestrial ecosystems^23–25^.

**Table 1:** Characteristics of fungal strains used in this work.

Strain code	Closest hit in NCBI database	% identity	Taxonomic Division	Growth rate (cm d^−1^)	
				on PDA	in Soil*
NFC-1	*Mortierella alpina*	99	Zygomycota	1.01 (0.01)	N/A
NFC-2	*Mortierella alpina*	99	Zygomycota	1.18 (0.01)	N/A
NFC-3	*Pleosporales sp*	99	Ascomycota	0.30 (0.05)	N/A
NFC-4	N/A	99	N/A	1.01 (0.01)	N/A
**NFC-5**	*Trichoderma atroviride*	99	Ascomycota	3.50 (0.02)	2.33 (0.13)
NFC-6	*Fusarium equiseti*	99	Ascomycota	1.50 (0.05)	0.23 (0.1)
NFC-7	N/A	N/A	N/A	2.80 (0.05)	N/A
NFC-8	N/A	N/A	N/A	3.38 (0.01)	N/A
NFC-9	N/A	N/A	N/A	3.11 (0.03)	N/A
NFC-10	*Aphanoascus terreus*	99	Ascomycota	0.50 (0.02)	N/A
NFC-11	N/A	99	N/A	0.30 (0.01)	N/A
NFC-12	N/A	99	N/A	0.67 (0.06)	N/A
NFC-13	*Phanerochaete magnoliae*	99	Basidiomycota	3.01 (0.01)	0.20 (0.11)
**NFC-14**	*Schizophyllum commune*	99	Basidiomycota	1.46 (0.01)	0.53 (0.04)
NFC-15	*Talaromyces purpureogenus*	99	Ascomycota	0.25 (0.02)	N/A
**NFC-16**	*Coprinellus radians*	100	Basidiomycota	1.64 (0.04)	0.41 (0.09)

Note: NFC-1 means the fungus-1 from Nazir Fungal Collection. The strains in bold were used for the soil microcosm experiments and further analyzed in terms of ARG-types, abundance and ARB numbers.

*The fungal growth in soil is corroborated here to visual observations of aerial mycelia of the respective fungus.

### ARB in fungal influenced microcosms

**Figure 2:**
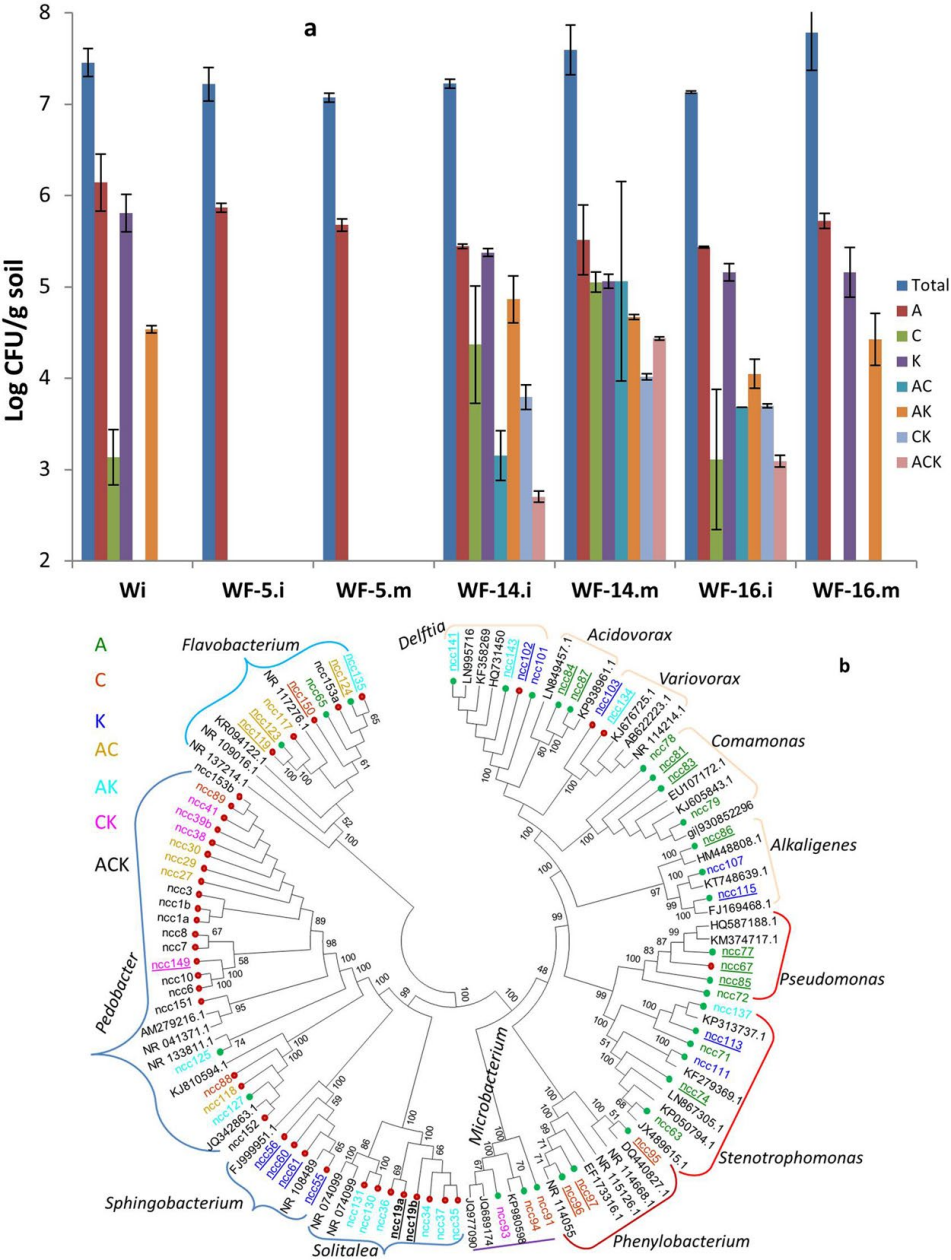
Cultivable ARB from the waste water W source affected or not by fungal colonization in pre-sterilized soil microcosms, sampled from inoculation point (i) and respective fungal migration front (m) (**a**), and their phylogenetic analyses (**b**). Blue bars are total bacteria on R2A without any antibiotic while A, C and K represent ampicillin, ciprofloxacin and kanamycin respectively, applied in medium alone or in combination. Error bars represent the standard deviations (n = 3). W corresponds to microbial inoculum originating from sludge/water of waste water treatment plant, while F-5, F-14, and F-16 represent the three selected fungal strains NFC-5, NFC-14, and NFC-16, respectively. *No bacteria were detected at migration point (Wm) for non-fungal treatments. In **‘b’** the strains with underline codes are isolated from m-point and others from i-point; Red and green nodes of the tree represent the M and W source respectively; while the text colors represent different antibiotics used to isolate these strains. The tree is constructed via neighbor joining method considering Maximum Composite Likelihood model with Bootstrap replicates of 1000, and bootstrap values equal to or greater than 50 are shown.

The microcosms were prepared with pre-sterilized Inner Mongolian soil, colonized or not with three fungal strains. The bacterial inocula for these experiments were wastewater treatment plant (W) and animal farm manure (M). A 3-comparment Petri plate was used as a microcosm with ‘Potato dextrose agar’ (PDA) as fungal growth medium in one section and test soil in the other two (Fig. 1). Fungus started growing in the PDA section and extended towards soil compartments where bacterial inoculation and subsequent samplings were carried out for various analyses. With the W inoculum, the total cultivable bacteria from the inoculation spot (i) in the fungal influenced treatments were >10^7^ CFU per gram of soil, when evaluated without any antibiotic in the R2A agar plates (control treatment) (Fig. 2a). Similar numbers (P > 0.05) were observed in the non-fungal (Wi) treatment. There were no bacteria detected at migration point (m) for non-fungal treatments. For the cultivable number of ARB from microcosms, ampicillin (A) resistant bacteria were present in all treatments with some minor fluctuations (nonsignificant differences, P > 0.05). Additionally, the ARB with other antibiotics (i.e. C, K, AC, AK, CK and ACK), were not supported in the mycosphere of NFC-5 either at the inoculation point (i) or migration front site (m). On contrary, the mycospheres of NFC-14 and NFC-16 enriched such ARB with significant fluctuations (P < 0.05) (Fig. 2a). For instance, the numbers of ARB with ciprofloxacin (C) resistance enriched by NFC-16 were similar to those in the inoculum (Wi) i.e. 10^3^ g^−1^ soil, increased up to two-fold by NFC-14, while not detectable in the NFC-5 mycosphere. Noticeably, the fungus NFC-14 not only supported ciprofloxacin resistant bacteria but also transported them in soil as they were detected as 10^5^ at the migration front (m) site (WF-14.m), while the situation was different with NFC-16 which supported these bacteria at the inoculation point but did not (or numbers under detection) transport (Fig. 2a). The same was true for the AC, CK and ACK sets of antibiotics for which the resistant bacteria were supported and transported by NFC-14, but not by NFC-16. Moreover, multi-antibiotics resistant bacteria at the inoculation point without any fungi (Wi) were not detected except for AK, but were preferentially enriched by the growing hyphal networks of NFC-14 and NFC-16. NFC-16 transported ARB for three out of seven antibiotics used, while NFC-14 helped to transport ARB for all the tested antibiotics. Furthermore, NFC-14 enriched the ARB along the transportation route on its mycelial networks e.g. ARB resistant to AC and ACK were 2-fold more in numbers at the migration front site (WF-14.m) compared to those present at the inoculation spot (WF-14.i).

Similar observations were recorded for the M inoculum originating from the animal farm-manure source (Fig. S1), except for detection of multi-antibiotic resistant ARB in the inoculum and comparatively reduced CFUs for all types of ARB at the migration front site (m) than at the inoculation spot (i) for NFC-14 which was different from W.

### Identification of cultivable ARB from fungal influenced microcosms

In total, 150 ARB strains were purified subsequent to the random selection (on colony morphology basis) from different fungal treatments using antibiotic treated agar plates (i.e. A, C, K, AC, AK, CK, ACK), among which 73 different morphotypes were subjected to 16 S rRNA gene sequencing for taxonomic identification (Table S1). Interestingly, 28 ARB strains belonged to *Proteobacteria* spp., 42 strains were *Bacteroidetes* spp. and three strains shared homology with *Actinobacteria* spp. (Fig. 2b). The majority of strains (88%) affiliated with *Bacteroidetes* spp. originated from the manure (M) source, while those with *Proteobacteria* spp. have mainly (86%) wastewater (W) origin. On the other hand, ARB strains isolated from the fungal migration front site (m) were spread across all taxonomic phyla. Noticeably, ampicillin (A) resistant bacteria were mainly confined to beta and gamma *Proteobacteria* spp. while ciprofloxacin (C) resistant bacteria mainly belonged to *Bacteroidetes* spp. along with *Actinomycete* spp. and alpha *Proteobacteria* spp. Kanamycin (K) and multi-antibiotic resistant bacteria were spread across phyla while broadly following the A and C trend (Fig. 2b). *Actinomycetes* spp. belonged to the *Microbacterium* genus containing C and CK resistant bacteria, originating from wastewater. In the *Bacteroidetes* spp., the major group belongs to the *Pedobacter* spp. ARB from manure (20/22). *Flavobacterium* spp. and *Solitalea* spp. contained eight ARB strains in each mainly originated from manure. All four members of the *Sphingobacterium* genus had K resistance and manure origin. On the other hand, *Stenotrophomonas* spp. (gamma proteobacteria) represented the major group containing six ARB originating from wastewater. *Pseudomonas* spp. (gamma-), *Comamonas* spp. (beta-) and *Delftia* spp. (beta- proteobacteria) had four ARB in each, representing ampicillin resistance for both. All these bacterial sequences were deposited in GenBank with accession numbers from MG193678 to MG193748.

### ARGs abundance and distribution in fungal colonized soil

**Figure 3:**
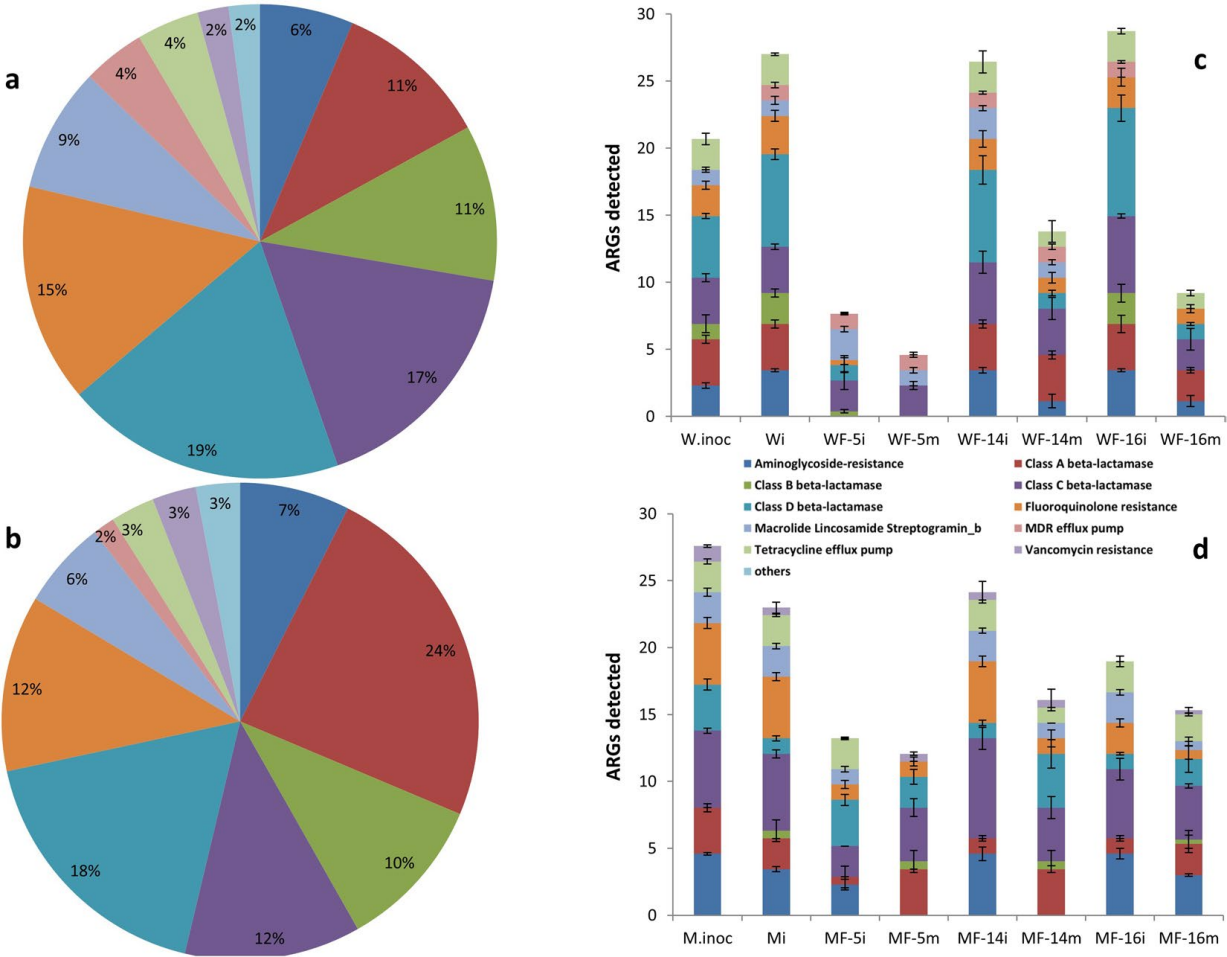
ARGs abundance (% representation of all detected ARGs) for different antibiotic resistance classes originating from waste water (**a**) and animal farm manure (**b**). Further numeric distribution of different ARG- classes of the W (**c**) and M (**d**) sources, influenced or not by different fungal infestation along the hyphal networks. Error bars represent the standard deviations (n = 3) for that particular ARG- class. W and M correspond to microbial inoculum originating from WWTP and manure respectively, while i and m represent the inoculation point and migration front respectively. F-5, F-14, and F-16 are the three selected fungal strains NFC-5, NFC-14, and NFC-16. *No ARGs were detected at migration point for non-fungal treatments (Wm and Mm).

The high-throughput qPCR assay was used to assess the fungal influence on ARB and subsequently the ARGs’ diversity and abundance through soil microcosm set ups. Any gene with C_T_ value detected below 36 was marked as positive. Out of 84 tested ARGs, the assay detected 27 and 20 different ARGs in M and W sources of inocula, respectively. The most abundant ARGs detected in the manure source (M) were class A beta lactamase (24%), class D beta lactamase (18%), class C beta lactamase (12%), quinolones (12%) and class B beta lactamase (10%), while it was class D beta lactamase (19%), class C beta lactamase (17%), qinolone (15%), class A and B beta lactamase (11% each) in the wastewater source (W) (Fig. 3a,b).

After 2-weeks incubation, we detected 27 ARGs with W inocula (Wi) (Fig. 3c) in non-fungal control treatments, while they decreased to 23 in the inoculation spot with M inocula (Mi) (Fig. 3d). The soil colonized by different fungal strains had significantly different effects on these ARG-types. For both M and W sources, NFC-14 sustained the detected number of ARGs at the control level, while NFC-5 significantly reduced to 13 and 7, respectively. Interestingly, all three fungi reduced ARGs along their hyphal networks, as the migration front (m) sites had a significantly lower number of ARGs compared to the respective inoculation (i) points, particularly with the wastewater source (Fig. 3c,d).

**Figure 4:**
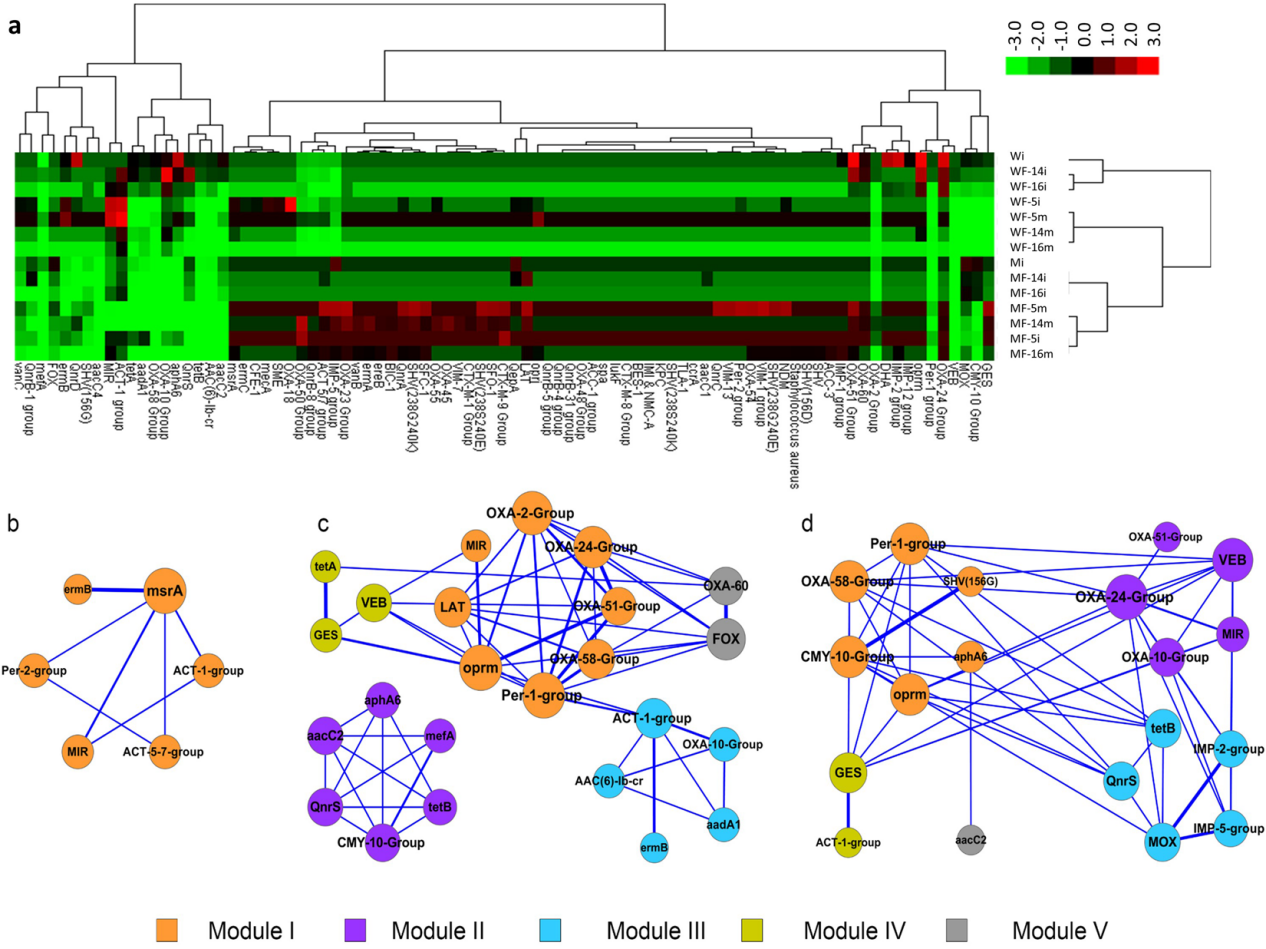
The heat map generated for different ARGs of the M and W sources under fungal influence, detected by qPCR assay (**a**), where each treatment- column represents three independent replicates. M, microbial inoculum originating from manure; W, microbial inoculum originating from sludge/water of waste water treatment plant; I, bacterial inoculation point; m, fungal migration front; 5, 14 and 16 are the selected fungi as in Table 1. The co-network analysis of different ARGs detected by qPCR assay, under the influence of fungal mycelial networks (n = 12) of NFC-5 (**b**), NFC-14 (**c**) and NFC-16 (**d**). The nodes with different colors (in b-d) represent different ARG- types detected by qPCR, and the edges correspond to a strong (*ρ* > 0.8) and significant (*P* < 0.05) correlation between nodes. The same color means the potential co-occurrence of ARGs in a same module while the size of each node is proportional to the number of significant connections.

Quantitative analysis for qPCR data was further performed via fold change, based on the comparative C_T_ method, considering the original inocula M and W, respectively, as the references for manure and wastewater sources of contamination (Fig. 4a). The heat map clustering produced four distinct groups, where firstly the treatments with the M inoculum were separated from those with the W inoculum. Interestingly, for both groups, the treatments from inoculation spot (i) were grouped together, except those colonized with the fungus NFC-5, which clustered with those from the fungal migration front site (m). In brief, fungus NFC-5 behaved differently to NFC-14 and NFC-16. Looking at the ARGs differentially affected by fungal colonization, the β-lactamases (e.g. OXA genes) were positively enriched by NFC-14 and NFC-16 for wastewater, and by NFC-5 for the manure source of contamination (Fig. 4a). The differential effect of these three fungi on different ARGs is briefly compiled in Figure S2. Similar to the heat map output, the principal component analysis also produced four different clusters based on the relative abundance of ARG types (Fig. S5). The treatments with manure inoculum (M) were separated from those with wastewater inocula (W). Additionally, the fungal transportation (migration) effect is also clearly depicted from such data. This differential fungal effect is exemplified by OXA-1, OXA-24 and ermB where NFC-5 behaved differently than the other two fungi along the mycelial networks from inoculation (i) points to the migration (m) fronts (Fig. S2).

### Co-occurrence network analysis of ARGs and spatial effect of fungal colonization in soil

Network analyses were conducted to explore the co-occurrence patterns of the detected ARGs after fungal colonization in manure and wastewater inoculated soil, based on strong (*ρ* > 0.8) and significant (*P* < 0.05) correlations. The fungal colonization had different effect on the co-occurrence networks at the inoculation (i) point compared to the ones they carried along their mycelia until the migration front (m) site (Fig. S3). For different fungi, the resulting networks (based on 12 technical replicates (3-biological replicates each for i and m points for M and W source contaminants) consisted of 6, 24 and 19 nodes (ARG types), while 7, 58 and 51 edges for fungi NFC-5, NFC-14 and NFC-16, respectively (Fig. 4b–d). The modularity indices, particularly for NFC-14 and NFC-16 were 0.512 and 0.692, respectively, which suggested that these networks had an obvious modular structure^26^ that could be separated into different modules. The ARGs within each module associated more frequently among themselves than with nodes in other modules, compared with random associations^27^. The co-occurring nodes confer resistance to the major types of antibiotics including aminoglycoside, various classes of β-lactam, fluoroquinolone and quinolone, MLS_b and vancomycin.

There were 1, 5 and 5 modules co-present in networks for NFC-5, NFC-16 and NFC-14, respectively (Fig. 4b–d). For example, the Per-1-group gene of class A β-lactam resistance as the hub (defined as the most densely connected node in the module) for NFC-14 included also the co-occurring OXA-genes of class D β-lactam resistance, which may indicate that these genes might be concomitantly harbored in some specific bacterial groups or some specific mobile genetic elements (even if in various microbial groups). Moreover, the modular structure of different ARGs’ co-occurrence was different at the migration front (m) compared to the inoculation (i) point (Fig. S3) which signified the fungal role (contribution) for the dissemination of ARGs along hyphal networks in soil.

### Transposon and integron abundance in fungal infested soil

**Figure 5:**
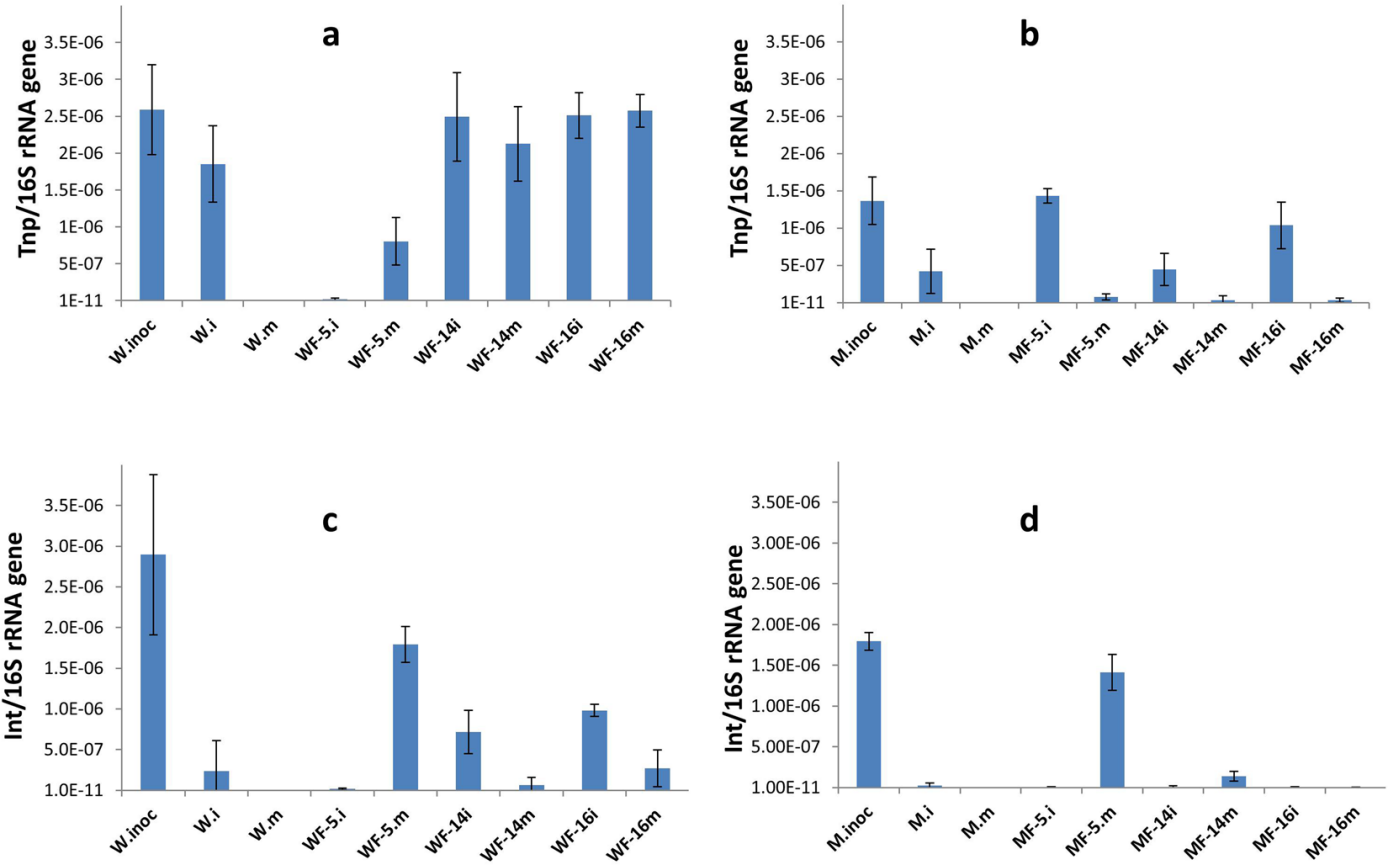
Relative abundance of transposon A (**a**,**b**) and integrase gene (**c**,**d**) for the W (**a**,**c**) and M (**b**,**d**) contamination sources, affected or not by colonization of different fungi in pre-sterilized soil microcosms. Error bars indicate standard deviations (n = 4). M.inoc, microbial inoculum originating from manure; W.inoc, microbial inoculum originating from sludge/water of waste water treatment plant; i, bacterial inoculation point; m, fungal migration front; 5, 14 and 16 are the selected fungi as in Table 1. Neither tnpA nor intI1 was detected for the treatments W.m and M.m while the detection limit for relative abundance of both was 1 × 10^−9^ gene copies per gram of soil.

To understand the horizontal gene transfer potential of ARGs across a bacterial community of a particular system it is important to estimate the relative abundances of transposons and integrons. The relative abundances of transposons (*tnpA*) and integrons (*intI1*) were calculated by normalizing the bacterial 16 S rRNA gene copies (Fig. S4) observed in the same sample via qPCR. Gene transfer factors i.e. *tnpA* and *intI1*, were detected in different treatments of the current fungal microcosm set ups (Fig. 5). The relative abundance of *tnpA* was observed to be around 10^−6^ in both the original contamination sources without fungal colonization, which remained the same level during the 2-weeks incubation for W source (W.inoc and W.i in Fig. 5a), while it significantly decreased for M source (M.inoc and M.i in Fig. 5b). However, with the influence of fungi on W source, NFC-14 and NFC-16 enriched similar level at inoculation point and migration front site which were significantly more than NFC-5. The relative abundance of *tnpA* were found under detection for NFC-5 at inoculation site but significantly enriched along its mycelia as found at migration front (Fig. 5a). For M source, all tested fungi enriched *tnpA* at inoculation point with no or marginal detection at migration front (Fig. 5b). As for integrons, the relative abundance of *intI1* gene decreased over the 2-weeks incubation either in the absence or presence of fungi (though significantly different for different fungi) for both contamination sources (Fig. 5c,d). Under the influence of fungal strains NFC-14 and NFC-16, the *intI1* gene abundance with W source showed a decreasing trend at the migration point compared to the inoculation point, while for fungal strain NFC-5 it was enhanced along the hyphal network, bringing the inoculum level to its migration front (Fig. 5c). Moreover, the relative abundance of *tnpA* and *intI1* genes was observed relatively less in M than respective W treatment (Fig. 5). Noticeably, the *intI1* gene trend along the hyphal networks of NFC-5 was similar to some ARGs (Fig. S2) i.e. decreasing at the inoculation (i) point compared to the inoculum and then increasing again at the migration front (m). Moreover, all three fungi supported significantly different levels of the *intI1* gene abundance at migration front (m) points, although originating from the same inoculum.

## Discussion

Soil represents one of the original habitats of antibiotics^28^, which has enabled soil bacteria to develop resistance and further adaptive strategies to successfully thrive in it. Our data demonstrated that fungal colonization in soil significantly influenced the distribution of ARB, the abundance of different ARGs, and the dynamics of HGT factors.

Amendment of soils with manure from antibiotic treated animals has extensively been studied as an important route by which ARGs enter the environment and food system^6,14,29–31^. Along with animal manure, wastewater from treatment plants, when used for irrigation purposes, is also recognized as an ARG-reservoir for environmental dissemination^3^. We used both contamination sources, and our data supported these entry pathways of ARGs through transmission factors (e.g. *tnpA*, *IntI1*), and the colonization of particular fungi consequently changed the selection pressure for ARB. Moreover, a source contaminant e.g. manure, may harbor distinct bacterial communities^32,33^ and our data are in line with this earlier finding, as bacteria isolated from manure and wastewater belonged mainly to *Bacteroidetes* and *Proteobacteria*, respectively (Fig. 2b). The antibiotic resistance was observed to be restricted to the taxonomic classification and even if HGT had played a role in ARGs dissemination, it is not the only case, at least not very frequently, with very divergent bacterial groups.

Importantly, the fungal colonization had a more pronounced effect than source contaminant on the establishment of soil bacterial community structures (Figs 2a and S1). Similar to the previous screening of soil ARGs^6,34^, genes conferring resistance to aminoglycoside, β lactam, quinolones and fluoroquinolones, and MLS_b were found in our samples which further indicated the potential fungal role in the distribution of ARGs in these earlier studies. Fluctuating abundances of detected ARGs in different mycospheres, irrespective of the source, possibly reflected the impact with which different soil colonizing fungi could differentially have, ultimately altering the soil reservoirs of ARGs. Noticeably, the provision of particular nutrients from growing fungi is thought to provide a competitive advantage to associated bacteria carrying ARGs e.g. β-lactamases^6^, and such bacterial growth enhancement by partner fungi has earlier been proved^21^. Our data affirmed both of these contentions as particular ARB were found to be preferentially increased in number along growing fungal hyphae (Fig. 2). The soil has earlier been recognized as a reservoir, among others, for broad spectrum β-lactamases^35,36^, which are commonly found^37^ in clinical settings^38^ as well. Our microcosms contained pre-sterilized soil, and no β-lactamase enrichment was observed without fungal colonization during experimental incubation for two different contamination sources; on the contrary, the reduced abundance from inoculation to migration spots in fungal infested systems indicated the specialized effect of fungal colonization on such ARGs.

Considering the mycosphere as a specialized bacterial habitat, it is well established that while growing, soil fungi can transport bacteria and chemical substances via their hyphal networks from one place in soil to another^12,39^. In this way, soil fungi can promote bioaccessibility for degradation of recalcitrant compounds^39^, oxalotrophic nutrition^40^, bacterial migration^41^ and mycorrhization helper effects^42^ among others. Our data aid ‘antibiosis’ as a new potential ecological role of soil growing fungi for preferential enrichment and dissemination of ARGs in soil. Therefore, this new insight regarding ecological functioning of soil fungi is very important to understand the problem of environmental ARGs, better risk assessment and relevant mitigation strategies.

Different microbial strategies can be postulated, which may dictate the fluctuations in ARB distribution and subsequently the ARGs abundance in fungal colonized (mycosphere) soil: (i) provision of specific energy compounds by fungi^21^ to specialized heterotrophic ARB harboring particular ARGs; (ii) fungal influence on the micro-environment for preferential bacterial survival^22^ and the stimulation of microbial associations through genetic interactions via influencing their cell competence^9^; (iii) production of specific antibacterial substances^43^ which target a set of bacteria, subsequently benefiting the others (the resistant ones); (iv) freely available soil DNA, e.g. as a result of cell lysis, can also be taken up by bacteria as well as by fungi^9^. Such hypothetical mechanisms may potentially contribute to the distribution of ARGs by fungal infestation via changing the diversity and abundance of ARB which host the resistance determinants. Moreover, some other factors e.g. heavy metals may also contribute to environmental ARGs^44,45^, but we overlooked this particular factor in our study to investigate the objective role of fungi.

In our study, the fluctuating abundance and diversity of ARGs’ under different fungal mycelial networks indicated the fungal role in the dispersal and horizontal transfer, which may subsequently influence the ARGs’ transfer to pathogenic bacteria. The differential selection of *intI1* and *tnpA* in different mycospheres (Fig. 5) may indicate the preferential selection of particular ARB which carry and transport different plasmids and ARGs in soils. Class 1 integrons have widespread distribution in Gram negative bacteria^46^, while *intI1* is reported to be very common in different environmental samples^47^. In an earlier work, a low abundance of ARGs was observed in the rhizosphere (with constant fluctuations) compared to the bulk soil, which suggested ongoing antibiotic resistance selection^15,48^. Such antibiotic resistance selection pressure could also be exerted via soil fungi, as in in the present study, which are integral rhizosphere components. For instance, for two of the three tested fungi the *intI1* abundance at the inoculation point was reduced compared to the non-fungal inoculum, and was even less at the migration front (Fig. 5), which may indicate the lower HGT frequencies, consequently lessening the potential of the occurrence and multiplication of ARGs (Fig. 3c,d). The opposite effect of the third fungus for enhanced *intI1* abundance along the migration front indicated the differential effect of different fungi on such genes and/or their host bacteria (ARB). Noticeably, the HGT rate in soil is assumed to be very low compared to the vertical ARGs transmission through ARB growth^5^. Therefore, such mechanisms^45^ still need more investigation in complex soil environments, particularly after the fungal colonization. The integrons have earlier been reported to be affected by soil type^14^, while different fungi are differentially present in soils and, thus, such observations should also be evaluated in the particular context of fungal colonization. Our current data demonstrated the differential effect of different fungi on the *intI1* and *tnpA* abundance, while network analyses further revealed the co-occurrence patterns of different types of ARGs in fungal treated microcosms. These co-occurring ARGs in each module might reside in the same mobile elements, bacteria or pathogens, which may have the potential to be transferred to humans under appropriate selection conditions, and thus require more work in future.

In conclusion, we demonstrated that soil colonizing fungi had clear impacts on the abundance and diversity of a broad spectrum of ARGs originating from two contamination sources. Although we agree that the pervasive antibiotic use is the most important factor for global ARGs, our results suggested that other factors including the soil inhabiting fungi can also contribute to this environmental phenomenon. As the current study was conducted in a pre-sterilized soil, further experiments should also be executed under natural conditions where the competition of inoculated bacteria and fungi with indigenous soil microbiota occurs, and where inoculation-source derived bacteria may have challenges to thrive^32,49^ by differentiating the environmental conditions^50^. In particular, the differential effect of fungal colonization of different strains in our study for ARGs and transmission factors (*intI1*, *tnpA*), emphasize the importance of differences between bulk soil and the rhizosphere^51^ (potentially containing fungi) that need to be considered when assessing the risks associated with ARGs spread. Therefore, future research is urgently needed to comprehensively assess the ARG- patterns in the context of bacterial-fungal consortia prevailing under different soil conditions, and to design options for minimizing subsequent dispersal of ARGs into the food chain.

## Material and Methods

### Sources of microbial inocula

#### Animal farm-manure

This source was collected from a pig farm from suburbs of Jiangsu province, China. The sample included animal feed, excreta and farm soil mixed with a ratio of 1:2:5 and was considered as a representative manure sample. This particular mixture was then suspended in sterilized water (1:15 wt/vol ratio), vigorously shaken on a vortex mixer (2–3 min) and subsequently on an orbital shaker for 10 min at 200 rpm. After shaking, the suspension was left standing for at least 15 min to settle soil particles and fungal spores, and was further filtered through a Whatman No. 1 paper. The filtrate was used as a bacterial inoculum (named as M) after 50× dilution with sterilized water.

#### Wastewater treatment plant

In order to use a source abundant in antibiotic resistance, the influent from the inlet of Qinghe Wastewater Treatment Plant, Beijing, China (40°02′N, 116°22′E) was collected. The sample was mixed well to homogenize the sludge with water and settled for 5–10 min, and the suspension above the settled sludge was considered as another bacterial inoculum (denoted as W) after 50× dilution with sterilized water.

### Isolation of saprotrophic fungi

Manure mixed with soil slurry (M) as mentioned above was used to enrich and isolate the most abundnant fungal species. The Potato Dextrose Agar (PDA) was prepared as 39 g l^−1^ (BD Difco) and supplemented with filter sterilized bacterial antibiotics including ampicillin (A) (Sigma Aldrich: broad spectrum ß-lactam antibacterial D-α-Aminobenzylpenicillin sodium salt), ciprofloxacin (C) (Sigma Aldrich: antibacterial 1-Cyclopropyl-6-fluoro-4-oxo-7-(piperazin-1-yl)-1,4-dihydroquinoline-3-carboxylic acid) and Kanamycin (K) (Sigma Aldrich: aminoglycoside antibiotic Kanamycin B sulfate salt) after sterilization. The M inoculum (without filtration) was serially diluted and 100 µl of each dilution was spread on the PDA plates with those antibiotics. The hyphal colonies were re-inoculated as fresh mycelia on new PDA plates to purify the fungal strains. The purified fungal strains were further analyzed for molecular identification via sequencing (with ITS4 primer) the PCR products of the ITS region amplified with ITS4 (5′-TCCTCCGCTTATTGATATGC-3′) and ITS5 (5′-GGAAGTAAAAGTCGTAACAAGG-3′)^52^. Consequently, the fungal sequences were deposited in GenBank with accession numbers from MG189945 to MG189961. On the basis of growth characteristics in our system (Table 1), three fast growing fungal strains were selected for further microcosm experimentation. Along with prevalence in the manure sample, *Trichoderma* spp. are normally present in all soils, frequently isolated as the most prevalent culturable fungi, opportunistic avirulent plant symbionts and saprophytes^24^. *Coprinellus* spp. and *Schizophyllum* spp. are basidiomycetous members of the *Agaricales* order, fungi which are widespread and prominent components of terrestrial ecosystems, performing a variety of ecological roles^25^.

The isolated fungal strains used in this study were further grown on the PDA plates for hyphal viability. Once every 4 weeks, the fungal strains were transferred to fresh PDA plates for maintenance. Furthermore, the fungal mycelia and spores were kept in glycerol as well as in sterilized water and as dry material for longer storage purposes.

### Soil microcosm set up

A grassland soil (pH 7.4 ± 0.4, sandy loam texture) collected from the Inner Mongolia region of China (42°02′N, 116°17′E) was used for soil microcosm construction. The soil was sterilized twice via autoclaving at 121 °C for 20 min each. Subsequent to 1^st^ autoclave, the soil was left at room temperature for at least 24 h before the 2^nd^ phase of sterilization. Soil sterility was checked by plating the slurries on the PDA as well as R2A plates and incubating at 28 °C. There was no bacterial or fungal growth on the plates even after a week, and so the soil was affirmed as sterilized. The microcosm system was established as reported previously^53^, consisting of Petri dishes with three compartments (Fig. 1). Two compartments were each filled with about 10–12 g of moist, sterilized soil (moisture contents corresponded to 60% of water holding capacity), yielding soil layers of about 4–6 mm in height. The third compartment of the microcosm was filled with PDA for fungal inoculation. The physical barriers between the compartments prevented compounds from the PDA to reach the soil compartments. These barriers were overcome by the fungal hyphae, and hence outgrowth of fungal mycelium from the nutrient-rich PDA into the soil was achieved (Fig. 1). This microcosm system was inoculated with different fungal strains (Table 1) on the PDA and incubated at 30 °C for 3–5 days before introducing bacterial inocula, thereby allowing the colonization of the PDA plus about 1–2 mm of the sterile soil. No fungi were inoculated in the control treatment. The bacterial cell suspensions (i.e. M and W) were separately added into the soil compartments by pipetting a 2–3 mm-wide stripe directly adjacent to the front of the growing fungal hyphae for the fungi inoculated treatment and in the similar place for the control treatment (Fig. 1). ‘Randomized complete block design’ (RCBD) was followed for microcosms sampling. Soil samples (approx. 100 mg) were taken from two sites within the soil compartments: i.e. the inoculation spot (named as point i) and the fungal migration front site (named as point m) after incubating the bacterial suspension for 15 days at 30 °C. These samples were used for DNA extraction and cultivation-based bacterial isolation. The microcosm experiments were independently conducted 3–4 times, providing the confidence for reproducibility of data regarding fungal influence.

### Colony-forming unit (CFU) counts and isolation of bacterial strains

Two technical replicates (as composite sample) and three independent biological replicates of each treatment were sampled and processed for subsequent analyses. The sampled soil was vigorously shaken in 0.85% NaCl buffer using a vortex mixer. Afterwards, the bacterial suspensions were diluted in a ten-fold series and subsequently plated (at least two dilutions for each sample) on the R2A plates supplemented or not with bacterial antibiotics. The antibiotics used in this study include Ampicillin (A), Ciprofloxacin (C) and Kanamycin (K) in sole or combinatorial fashion, resulting in the following treatments: A, C, K, AC, AK, CK and ACK. CFU counts on all plates were enumerated following incubation for 2–5 days at 30 °C. 2–3 plate counts for each condition were recorded to have comparatively consistent numbers for data compilation. Moreover, bacterial colonies were randomly picked for each treatment and streaked to purity. Subsequently, the bacterial isolates were subjected to presumptive identification by their 16 S rRNA gene sequence, as per standard techniques^54^.

Bacterial strains, on purity, were grown in LB, mixed with sterilized 30% glycerol, snapshot frozen with liquid nitrogen and stored at −80 °C as basic stock. For regular use, bacteria were grown on LB and/or R2A for maximally four transfers after the culture was retrieved from the −80 °C stock.

### The isolation of total genomic DNA

Total DNA was extracted from 0.5 g of sampled soil by using MoBio PowerSoil DNA Isolation kit (MoBio Laboratories, Carlsbad, CA, USA) *as per* the manufacturer’s instructions, with slight modifications to the initial cell lysis step by using a FastPrep bead beating system (Bio 101, Vista, CA, USA) at a speed of 5.5 m s^−1^ for 30 s. The DNA concentration and quality were assessed using a NanoDrop ND-1000 Spectrophotometer (NanoDrop Technologies, Wilmington, DE, USA). For the majority of the soil DNA extracts, the A260/A280 ratios were greater than 1.8 which indicated a good extraction procedure.

### High throughput profiling of ARGs by quantitative PCR (qPCR) arrays

The occurrence and fungal affected changes of ARGs were analyzed using the Antibiotic Resistance Genes Microbial DNA qPCR arrays (Qiagen, Valencia, CA, USA) according to the manufacturer’s instructions, enabling high PCR specificity and accurate quantification. This array can simultaneously target a broad spectrum profile of 84 ARGs from all major classes, including aminoglycoside, β lactam (classes A, B, C, and D), erythromycin, quinolone and fluoroquinolone, marcrolide lincosamide streptogramin_b (MLS_b), tetracycline, vancomycin, and multidrug resistance. These targeted ARGs are associated with intensively used antibiotics in veterinary as well as human medication. The reaction mixture consisted of 25 µl including 12.5 µl HotStart DNA Polymerase Mastermix (Qiagen), 5 ng template DNA, and DNA free water to make up the volume. The reaction mixtures were aliquoted into each well of the 96 well array plate, containing a fluorescent hydrolysis probe and a mix of two pre-dispensed, gene specific primers. Pan bacterial assays were included as positive controls for the presence of bacterial DNA, and the positive PCR control assay was also included to test for PCR inhibitors and the efficiency of qPCR runs using a pre-dispensed artificial DNA sequence and the primers that detect it. Thermal cycling was performed on an iCycler iQ5 Thermocycler (Bio Rad, Hercules, CA, USA) as follows: an initial PCR activation step at 95 °C for 10 min, followed by 40 cycles of 15 s at 95 °C for denaturation and 2 min at 60 °C for annealing and extension. The baseline and threshold fluorescence values were manually adjusted to the same levels across all qPCR runs, and a threshold cycle (C_T_) value of 36 was used as the detection limit, as defined earlier^6^. The C_T_ values generated from the qPCR runs were imported into the Data Analysis Template Excel Software (Qiagen) to calculate the fold change values of all ARGs. The C_T_ method of relative profiling was conducted, with three replicates, to evaluate the ARGs dynamics in soil samples as described earlier^6,30^.

### qPCR analysis of the *intI1*, *tnpA*, and bacterial 16 S rRNA genes

The abundances of the *intI1* gene (an integrase gene of class 1 integrons) and the *tnpA* gene (a transposase gene) were quantified on an iCycler iQ5 Thermocycler (Bio Rad) using the primer sets HS463a/HS464^55^ and tnpA-04F/tnpA-04R^30^, respectively. The total bacterial 16 S rRNA gene was also quantified using the BACT1369F/PROK1492R with the probe TM1389F^56^. Amplification was performed in a total volume of 25 µl including 12.5 µl of SYBR Premix Ex Taq (TaKaRa Biotechnology, Otsu, Shiga, Japan), 1 µl of each primer (10 µM), and 1 µl of template DNA. For quantification, standard curves were generated via preparing 10-fold serial dilutions of plasmids containing correct inserts of the target genes. The specificity of PCR amplicons was verified by melting curve analysis following each qPCR run. PCR efficiency ranged from 85 to 96% for all the qPCR runs. The relative abundance of the *intI1* and *tnpA* genes was calculated by normalizing to the bacterial 16 S rRNA gene abundance to compensate for variance induced by differential DNA extraction efficiency and amplification efficiency across samples.

### Co-occurrence network analysis and visualization

The co-occurrence patterns between different ARG types detected with the high-throughput qPCR array were explored in network analysis using the Cytoscape plug-in CoNet^57,58^. Briefly, for all the pairwise interactions, correlation scores were calculated using Pearson correlation, Spearman correlation, mutual information, Bray Curtis dissimilarity, and Kullback Leibler dissimilarity. All ARG types below a minimum occurrence of 3 across all the samples (considering 12 replicates for each tested mycosphere) were discarded to avoid introduction of spurious correlations. The ReBoot procedure with 100 permutations was conducted to control the potentially false positive correlations, and the resultant distribution was further refined with 1000 bootstraps. The P values for correlations were combined from the five correlation measures using the Brown method, and only correlations found to be significant by at least two correlation methods were included^59^. The resultant pairwise correlations between the ARG types were utilized to construct their co-occurrence networks^60^. Only correlations with a ρ value above 0.8 and a significance level below 0.05 were displayed^61^.

### Statistical analysis

Soil sampling from microcosms was performed according to the randomized complete block design (RCBD). Replicated samples (at least 4 replicates for cultivation based analyses and 3 replicates for qPCR assays) were taken from two different places of the soil compartments. The microcosm experiments were independently conducted 3–4 times, providing the confidence for reproducibility of data regarding fungal influence. The CFU counts for antibiotic resistant bacteria (ARB) and relative abundances of the *intI* and *tnpA* genes were log transformed prior to statistical analyses to meet normality assumptions. Linear regression analysis was conducted to relate the relative abundance of the *intI* and *tnpA* genes to the fold change values of ARGs based on the log transformed data. Statistically significant differences were accepted at *P* < 0.05. The heat maps illustrating the qPCR array results of ARGs with log transformed fold changes were generated using the gplots package in R.3.2.5. All the tests were performed at least in triplicates and results are presented as averages.

Variations in data were observed using standard deviations which are shown as error bars in graphs and numerical values in the text.

## Electronic supplementary material

Supplementary material

Supplementary Table 1
