# Supplementary material for: Fungal networks serve as novel ecological routes for enrichment and dissemination of antibiotic resistance genes as exhibited by microcosm experiments
Source: Sci Rep. 2017 Nov 13;7:15457. doi: 10.1038/s41598-017-15660-7 (PMC5684214; doi:10.1038/s41598-017-15660-7)

Fungal networks serve as novel ecological routes for enrichment and dissemination of antibiotic resistance genes as exhibited by microcosm experiments

Rashid Nazir[1,2], Ju-Pei Shen[1*], Jun-Tao Wang[1], Hang-Wei Hu[3], Ji-Zheng He[1,3*]

[1] *State Key Laboratory of Urban and Regional Ecology, Research Centre for Eco-Environmental Sciences, Chinese Academy of Sciences, Beijing 100085, China.*

[2] *Department of Environmental Sciences, COMSATS Institute of Information Technology, Abbottabad 22060, Pakistan.*

[3] *Faculty of Veterinary and Agricultural Sciences, The University of Melbourne, Parkville, Victoria 3010, Australia.*

*Author for correspondence: Ju-Pei Shen, email: jpshen@rcees.ac.cn OR Ji-Zheng He, E-mail: jzhe@rcees.ac.cn, Tel: (+86) 10 62849788, Fax: (+86) 10 62923563

Figure S1 Fungal effect on the culturable ARB from the animal manure (M) source, sampled from inoculation point (i) and respective fungal migration front (m). F-5, F-14, and F-16 represent the three selected fungal strains NFC-5, NFC-14, and NFC-16, respectively. Blue bars are total bacteria on R2A without any antibiotic while A, C and K represent ampicillin, ciprofloxacin and kanamycin respectively, applied in medium alone or in combination. *No bacteria were detected at migration point (Mm) for non-fungal treatments.

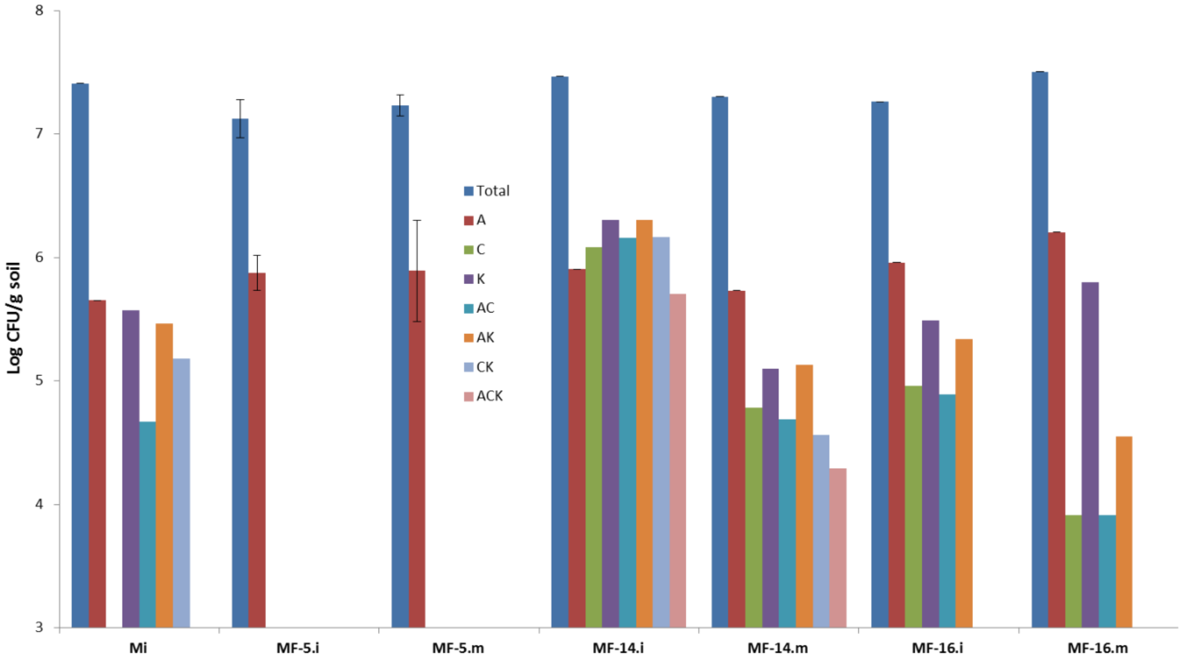

Figure S2 Effect of different fungi on (selected) ARGs abundance, relative to the original W inoculum. F-5, F-14, and F-16 represent the three selected fungal strains NFC-5, NFC-14, and NFC-16, respectively. i and m are the bacterial inoculation point and fungal migration front respectively.

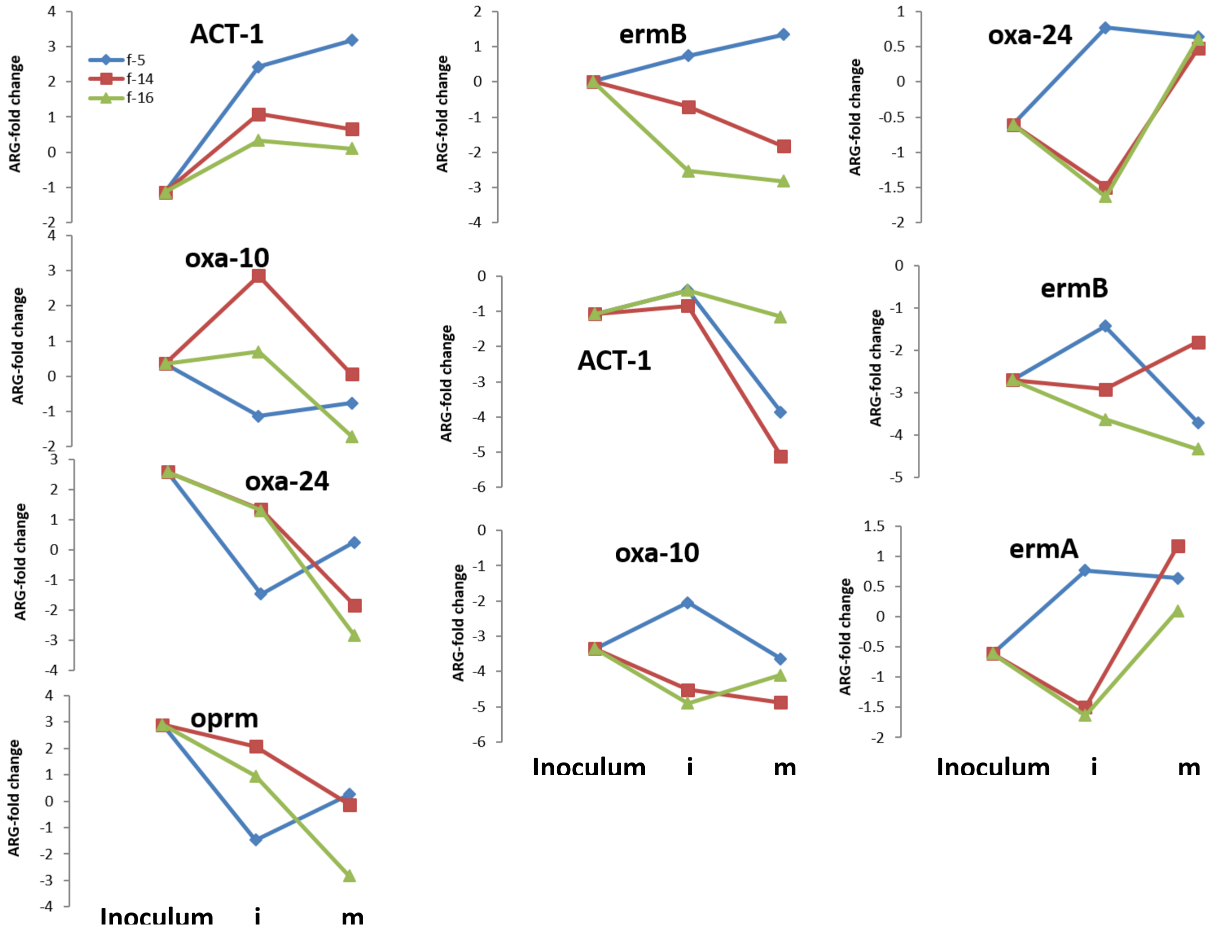

Figure S3 Fungal colonization effect and ARGs- network analysis for inoculation point **(a)** and migration front **(b).** The nodes with different colors (in b-d) represent different ARG-types detected by qPCR, and the edges correspond to a strong ($\rho > 0.8$) and significant ($P < 0.05$) correlation between nodes. The same color means the potential co-occurrence of ARGs in a same module while the size of each node is proportional to the number of significant connections.

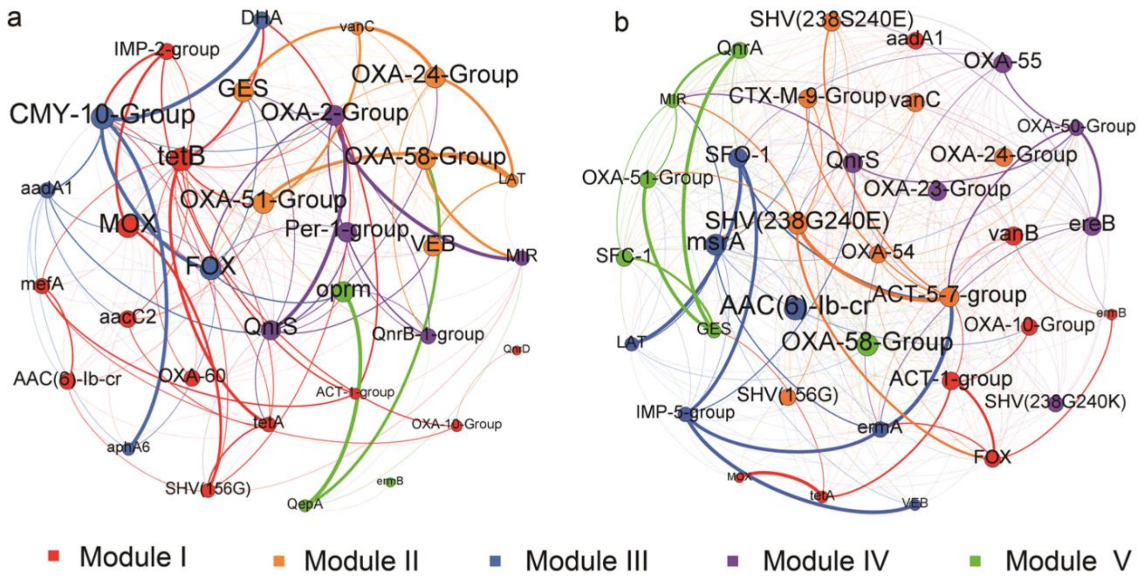

Figure S4 The bacterial 16S rRNA gene abundance in different soil samples affected or not with fungal colonization. M, microbial inoculum originating from manure; W, microbial inoculum originating from sludge/water of waste water treatment plant; i, bacterial inoculation point; m, fungal migration front; 5, 14 and 16 are the selected fungi as in table 1.

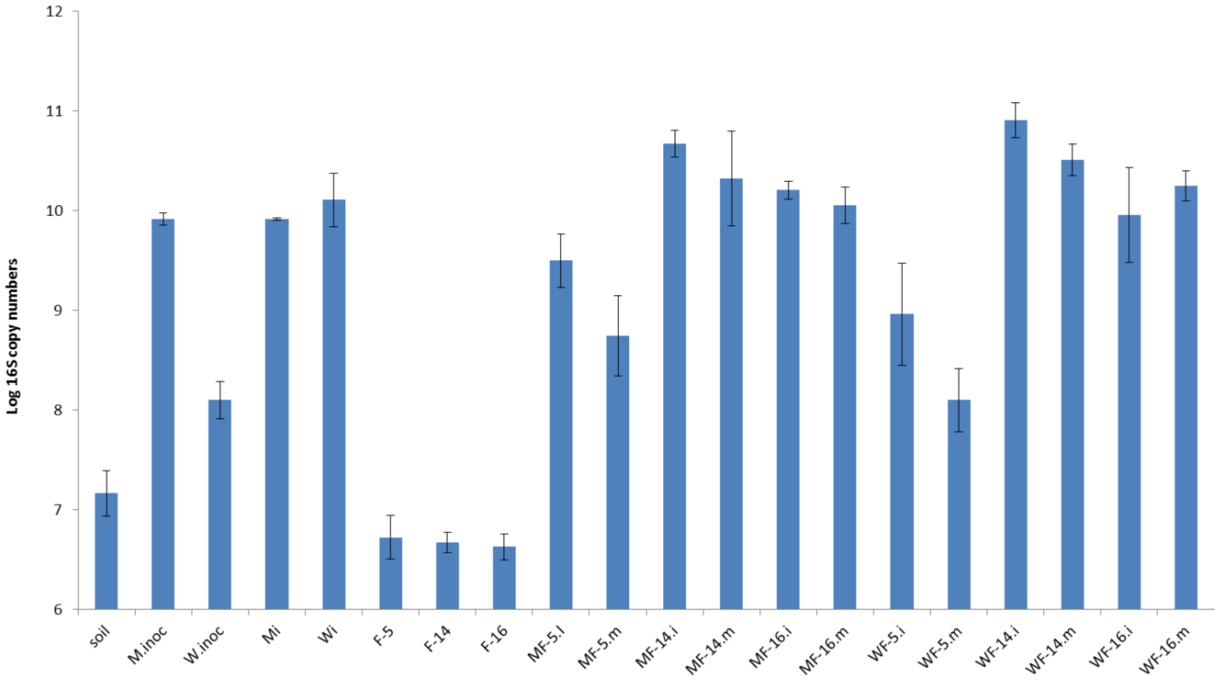

Figure S5 Principle component analysis for bacterial ARGs of W and M source, influenced by fungal colonization. qPCR data for 84 ARGs were used for each treatment to calculate PC1 and PC2. Bi means bacterial inoculum either from manure (M) or waste water (W), i means inoculation point in soil microcosm and m means the fungal migration front.

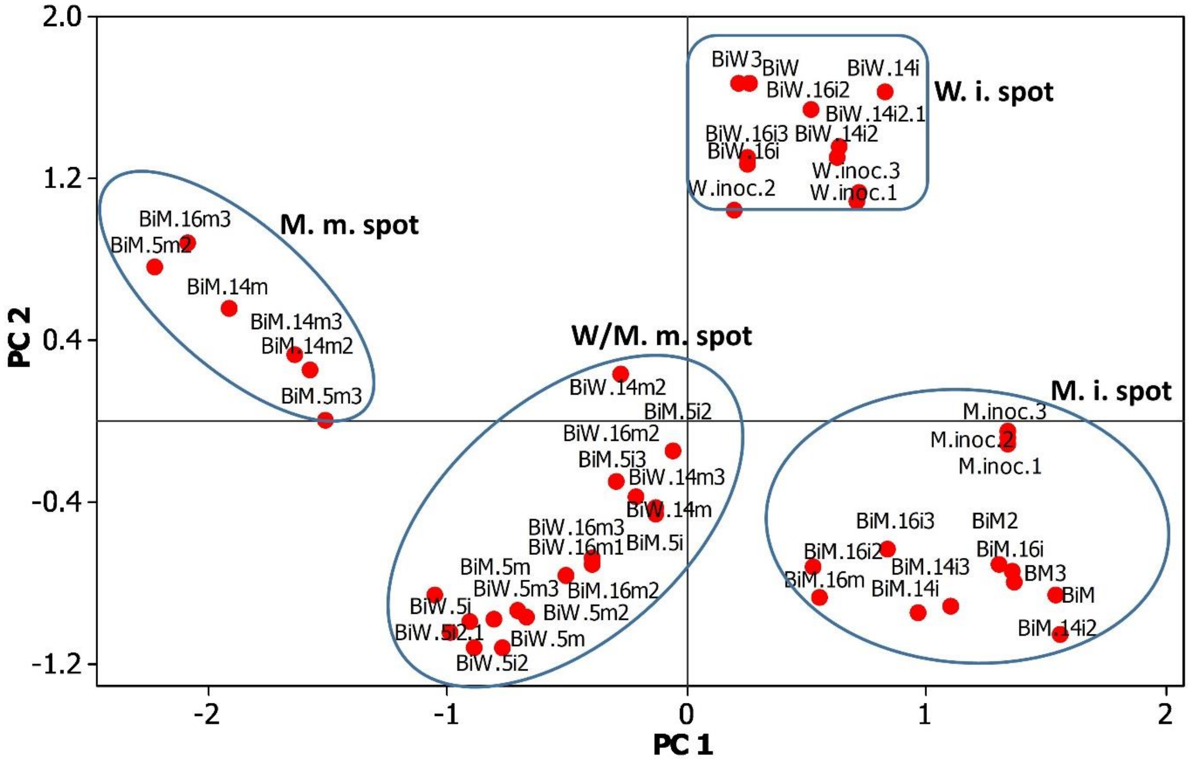

Supplement: Supplementary file 1 — Supplementary material [file 41598_2017_15660_MOESM1_ESM.pdf]
